# CRP-derived lower-limb coordination pattern distributions and frequency-band trunk–lower limb intermuscular coherence during badminton backhand footwork: a cross-sectional study

**DOI:** 10.3389/fphys.2026.1922514

**Published:** 2026-10-21

**Authors:** Hongbing Xie, Hangyuan Li

**Affiliations:** 1 Department of Physical Education, Xinjiang Second Medical College, Karamay, China; 2 Department of Basic Education, Xinjiang Institute of Political Science and Law, Tumushuke, Xinjiang, China

**Keywords:** backhand footwork, badminton, intermuscular coherence, joint coordination, surface electromyography

## Abstract

**Objective:**

To compare complete-cycle lower-limb coordination-pattern distributions and frequency-band-specific trunk–lower limb intermuscular coherence during a standardized badminton backhand footwork–backhand-clear task between elite and amateur players.

**Methods:**

Thirty-six male badminton players were classified into elite and amateur groups (n=18 each). Each participant completed 10 valid trials. Three-dimensional kinematics and surface electromyography were synchronously recorded using Qualisys and Delsys Trigno systems. Continuous relative phase waveforms were summarized as four coordination-pattern percentages for the hip–knee, hip–ankle, and knee–ankle pairs. Intermuscular coherence was quantified as the area of significant coherence (AZ) for 21 trunk–lower limb muscle pairs across α, β, and γ bands. Trial-level linear mixed-effects models included group, trial, and their interaction as fixed effects and participant as a random intercept. Years of badminton training were not included as a covariate, as the objective was to compare competitive-level groups within their existing training backgrounds. Benjamini–Hochberg false discovery rate (FDR) correction was applied separately to 12 coordination and 63 coherence comparisons.

**Results:**

After FDR correction, elite players showed higher hip–knee in-phase and lower anti-phase proportions, higher proximal-dominant and lower distal-dominant proportions for hip–ankle and knee–ankle coordination, and a higher knee–ankle anti-phase proportion (all q=0.025). Although 23 coherence comparisons had raw P<0.05, none survived FDR correction (all q≥0.098).

**Conclusion:**

Elite and amateur players differed in selected complete-cycle coordination-pattern distributions, with greater proximal contribution to hip–ankle and knee–ankle coordination in elite players. No muscle-pair–frequency-band coherence difference remained significant after FDR correction. These task-specific, cross-sectional findings do not identify the timing of coordination differences, establish causal effects of training, or demonstrate competitive-level associations independent of training duration.

## 1 Introduction

Badminton is a high-intensity intermittent racket sport that requires rapid court coverage, repeated acceleration and deceleration, multi-directional footwork, and precise stroke execution under time pressure (Shaharudin et al., 2026). Among badminton techniques, the backhand stroke is often performed in a relatively passive rear-court situation, where players must quickly move toward the non-racket side, establish stable lower-limb support, rotate the trunk, and complete an effective stroke within a short time window (Steels et al., 2020). Although upper-limb racket movement directly determines shuttle contact, successful backhand execution largely depends on whether the player can reach the optimal hitting position through efficient footwork (Marchena-Rodriguez et al., 2024). Therefore, the badminton backhand footwork technique (BBFT) represents a key sport-specific movement that integrates rapid lower-limb displacement, braking support, postural control, and stroke preparation. BBFT is important for players of different competitive levels because it determines not only movement efficiency but also the quality and stability of subsequent stroke execution (Wang et al., 2024).

The biomechanical basis of BBFT is mainly reflected in lower-limb joint movement and neuromuscular control. During rear-court backhand movement, the hip, knee, and ankle joints must coordinate to complete propulsion, lateral-backward displacement, braking, and support before racket–shuttlecock contact (Hung et al., 2020). Previous badminton studies have examined footwork speed, lower-limb kinematics, ground reaction force, muscle activation, and fatigue-related movement changes (Phomsoupha and Laffaye, 2015). These studies have shown that higher-level players usually demonstrate faster movement, better braking ability, and more stable lower-limb control during sport-specific footwork (Lee and Loh, 2019). However, most existing studies have focused on isolated joint angles, peak force, movement time, or single-muscle activation. Such variables cannot fully explain how adjacent joints coordinate across the complete movement cycle or how trunk and lower-limb muscles interact during BBFT (Hung et al., 2020). Therefore, the coordination mechanism underlying elite backhand footwork remains insufficiently clarified.

Lower-limb joint coordination is essential for efficient badminton footwork because sport-specific movement is not produced by a single joint but by the continuous coupling of multiple joints within the kinetic chain (Lu et al., 2022). Continuous relative phase analysis can describe the phase relationship between joints and identify coordination patterns such as in-phase, anti-phase, proximal-dominant, and distal-dominant patterns (Li et al., 2025). This approach may reveal whether elite players rely more on proximal control or distal compensation during rapid braking and stroke preparation. In addition, intermuscular coherence provides information about common neural drive and functional coupling between muscles in specific frequency bands (Zhuang et al., 2026). Previous neuromuscular studies have suggested that α-, β-, and γ-band coherence may reflect different aspects of sensorimotor integration, corticospinal modulation, and task-related motor coordination (Liang et al., 2020). However, limited evidence has examined trunk–lower limb time–frequency intermuscular coherence during badminton-specific footwork, especially in BBFT.

Comparing elite and amateur players provides a useful framework for identifying coordination characteristics associated with badminton expertise. Although both groups are able to perform the fundamental BBFT movement, they differ in systematic training exposure, competitive experience, and technical proficiency. Long-term participation in structured badminton training may be associated with more stable inter-joint organization, more effective proximal-to-distal control, and different patterns of common neural input between the trunk and lower-limb muscles. However, these expertise-associated differences cannot be inferred from isolated joint angles, movement time, or single-muscle activation alone.

A direct comparison between elite and amateur players may therefore clarify whether higher competitive status is associated with distinct complete-cycle lower-limb coordination-pattern distributions and frequency-band-specific trunk–lower limb coupling during the same standardized BBFT task. Identifying such characteristics may provide objective reference information for future longitudinal studies of badminton training and athlete development. Nevertheless, because the present study used a cross-sectional design, the comparison was intended to identify associations with competitive level rather than to establish that competitive level or training experience caused the observed coordination differences.

Accordingly, this cross-sectional study compared elite and amateur badminton players during a standardized BBFT task using synchronized three-dimensional motion capture and wireless surface electromyography. Lower-limb coordination was quantified using the complete-cycle percentages of in-phase, anti-phase, proximal-dominant, and distal-dominant CRP patterns for the hip–knee, hip–ankle, and knee–ankle joint pairs. Trunk–lower limb neuromuscular coupling was quantified using frequency-band-specific AZ values for 21 muscle pairs. The study aimed to determine whether elite and amateur players differed in their overall lower-limb coordination-pattern distributions and frequency-specific intermuscular coupling during BBFT. We hypothesized that the two groups would demonstrate different complete-cycle coordination-pattern distributions and distinct frequency-band-specific trunk–lower limb coupling characteristics.

## 2 Methods

### 2.1 Participants

Participants were classified into the elite and amateur groups using prespecified criteria based on official athlete classification, competitive experience, and participation in systematic sport-specific training. The elite group consisted of players who had received systematic badminton training within a structured competitive program, held a valid National Level-2 Athlete certificate or higher, and had participated in provincial-level or higher badminton competitions. The amateur group consisted of recreationally trained players who had at least 2 years of regular badminton practice and were able to perform the standardized backhand footwork and rear-court backhand clear independently. Amateur participants did not hold a National Level-2 Athlete certificate or higher, had not achieved a top-three result in a provincial-level or higher competition, and were not currently registered as competitive badminton athletes. “National Level-2 Athlete” refers to an official athlete classification within China’s national athlete technical grading system. The certificate is awarded by an authorized sports administration body on the basis of competition results that meet the applicable sport-specific grading standards. In the present study, elite participants held a National Level-2 Athlete certificate or higher and had achieved a top-three placing in provincial-level or higher badminton competitions.

An *a priori* sample size calculation was performed using G*Power version 3.1 (Heinrich Heine University Düsseldorf, Düsseldorf, Germany). The calculation was based on the planned two-sided comparison between two independent groups, with an allocation ratio of 1:1. Based on the effect observed in the preliminary testing, a large standardized between-group effect of Cohen’s d = 1.00 was assumed for the primary lower-limb coordination outcome. With a two-sided significance level of α = 0.05 and a statistical power of 1 − β = 0.80, the minimum required sample size was 34 participants, corresponding to 17 participants per group. To account for approximately 5%–6% potential data loss resulting from invalid trials, marker detachment, poor-quality EMG signals, or synchronization failure, 36 participants were recruited. The final sample therefore consisted of 18 participants in the elite group and 18 participants in the amateur group, exceeding the minimum required sample size. The sample size calculation did not incorporate the potential gain in precision from the repeated trials included in the subsequent mixed-effects analysis and was therefore used as a conservative planning estimate for the primary between-group comparison.

Years of badminton training were defined as the total number of years during which a participant had engaged in regular, structured badminton practice. This variable was obtained from the participant’s training history and verified, where available, using athlete-registration, certification, or team-training records. Years of training were recorded for all 36 participants, reported separately for the elite and amateur groups in **Table 1**,

**Table 1:** Basic characteristics of the participants.

Variable	Elite group (n = 18)	Amateur group (n = 18)	t	P	Cohen’s d
Age (years)	22.4 ± 2.1	21.8 ± 1.9	0.90	0.375	0.30
Height (cm)	178.6 ± 5.7	176.9 ± 6.1	0.86	0.394	0.29
Body mass (kg)	72.4 ± 6.8	70.9 ± 7.2	0.64	0.525	0.21
BMI (kg/m²)	22.7 ± 1.8	22.5 ± 2.0	0.32	0.754	0.11
Badminton training experience (years)	8.6 ± 2.4	4.3 ± 1.5	6.45	<0.001	2.15

The inclusion criteria were as follows: (1) aged 18–30 years; (2) right-handed or with a clearly defined dominant racket-holding side; (3) having clear badminton experience and being able to independently and stably perform the standard backhand footwork technique; (4) continuously participating in badminton training or badminton activities within the past 6 months; (5) being able to understand the experimental procedures and complete all testing tasks; and (6) voluntarily providing written informed consent.

The exclusion criteria were as follows: (1) acute injury to the lower limbs, trunk, shoulder, elbow, or wrist within the past 6 months; (2) severe muscular, skeletal, or joint disorders that could affect the performance of the backhand footwork technique; (3) neurological disorders, vestibular dysfunction, or other conditions affecting motor control; (4) high-intensity training, formal competition, or obvious fatigue within 24 h before testing; and (5) inability to complete the prescribed task, obvious movement deformation, abnormal electromyographic signals, motion-capture marker detachment, or synchronization failure during testing.

This study was approved by the Ethics Committee of Xinjiang Second Medical College (Ethics approval number: XSMC20260131) and was conducted in accordance with the Declaration of Helsinki. Before testing, all participants were informed of the study purpose, procedures, precautions, and potential risks, and voluntarily signed written informed consent. Participants were required to avoid high-intensity training, formal competition, alcohol consumption, and other behaviors that could affect neuromuscular function and motor performance within 24 h before testing.

### 2.2 Experimental design

This study adopted a cross-sectional comparative design, with the badminton backhand footwork technique used as the testing task. The study compared movement performance, lower-limb joint coordination characteristics, and trunk–lower limb time–frequency intermuscular coherence between the elite and amateur groups under the same sport-specific movement condition. During testing, three-dimensional kinematic data and surface electromyographic signals were synchronously collected using an infrared three-dimensional motion capture system and a wireless surface electromyography system while participants performed the backhand footwork technique.

The main outcome measures included three categories. First, movement performance outcomes included backhand footwork completion time and shot-placement accuracy. Second, lower-limb joint coordination outcomes included the distribution of coordination patterns for the hip–knee, hip–ankle, and knee–ankle joint pairs. Third, neuromuscular coordination outcomes included time–frequency intermuscular coherence of trunk–lower limb muscle pairs across different frequency bands. Athlete level was used as the between-group factor to compare differences in movement performance, lower-limb joint coordination, and trunk–lower limb neuromuscular coordination characteristics between the elite and amateur groups during the backhand footwork technique.

#### 2.2.1 Testing instruments

An infrared three-dimensional motion capture system was used to collect kinematic data during the backhand footwork technique. The motion capture system was a Qualisys three-dimensional motion capture system (Qualisys AB, Gothenburg, Sweden), with the sampling frequency set at 200 Hz. This system was used to record the three-dimensional trajectories of the pelvis, hip joint, knee joint, ankle joint, and foot during the backhand footwork technique, and to calculate hip, knee, and ankle joint angles, angular velocities, and joint coordination indices in subsequent analyses. Before formal testing, spatial calibration was performed according to the system operating procedures to ensure the accuracy of kinematic data acquisition.

Surface electromyographic signals were collected using a wireless surface electromyography system, namely the Delsys Trigno Wireless EMG System (Delsys Inc., Natick, MA, USA). The sampling frequency was set at 1000 Hz, and the signal acquisition bandwidth was 20–450 Hz. This system was used to synchronously record surface electromyographic signals from the target lower-limb and trunk muscles during the backhand footwork technique and to provide raw data for subsequent trunk–lower limb time–frequency intermuscular coherence analysis.

To ensure temporal consistency between the kinematic data and surface electromyographic signals, an external synchronization trigger system was used to synchronize the infrared three-dimensional motion capture system and the wireless surface electromyography system. Before formal testing, a common time origin for the two systems was established through an external trigger interface. During offline analysis, the synchronization trigger signal was used as the common temporal reference, and surface electromyographic signals within the corresponding movement cycle and movement phases were extracted and analyzed based on the key movement events identified from the motion capture data.

All tests were conducted on a standard badminton court. Before testing, the central starting position, rear-court backhand hitting area, and target landing area were uniformly marked to ensure consistent testing conditions across participants. The target landing area was set in a designated area of the opposite court and was used to record shot-placement outcomes and calculate shot-placement accuracy.

#### 2.2.2 Definition and standardization of the backhand footwork task

The testing task consisted of a standardized badminton backhand footwork technique (BBFT) followed by a rear-court backhand clear. Participants started from the central ready position, moved rapidly toward the designated rear-court backhand area on the non-racket side, established a stable supporting position, and executed a backhand clear toward a predefined target area in the opponent’s rear court.

The backhand clear was selected as the standardized stroke for all participants. Participants were instructed to strike the shuttlecock with a high and deep trajectory toward the opponent’s rear court. Backhand drops, backhand smashes, drives, slices, and other backhand stroke variations were not permitted. The complete task included movement initiation, backward-lateral displacement, rear-court braking and support, trunk rotation, backhand-clear execution, and follow-through recovery.

A trial was considered valid when the participant initiated movement in the prescribed direction, completed the required footwork without interruption, reached the designated rear-court hitting area, contacted the shuttlecock using a backhand-clear action, and directed the shuttlecock toward the opponent’s rear court. Trials were excluded and repeated if the participant used another stroke type, failed to reach the prescribed hitting area, performed an incomplete stroke, lost balance, or experienced marker detachment, abnormal EMG signals, or synchronization failure (Tian et al., 2025).

To ensure consistent temporal boundaries for kinematic and time–frequency intermuscular coherence analyses, the BBFT movement cycle was uniformly defined. The BBFT analysis cycle was defined as the period from movement initiation at the central ready position to racket–shuttlecock contact. Movement initiation was defined as the instant when the support foot or moving foot began to clearly leave the ground, or when the body center of mass began to move toward the backhand side. Racket–shuttlecock contact was defined as the instant when the racket contacted the shuttlecock. According to the technical structure and functional characteristics of BBFT, the complete movement cycle was further divided into three phases: the movement initiation phase, braking support phase, and stroke force-generation phase. The movement initiation phase was defined as the period from movement initiation to before initial contact of the main support foot, mainly reflecting the ability to move rapidly from the central position toward the rear-court backhand area. The braking support phase was defined as the period from initial contact of the main support foot to the completion of body center-of-mass braking and preparation for stroke execution, mainly reflecting lower-limb braking, support stability, and postural control. The stroke force-generation phase was defined as the period from the end of braking support to racket–shuttlecock contact, mainly reflecting coordination among lower-limb support, trunk control, and backhand stroke execution. All key movement events were identified offline based on infrared three-dimensional motion-capture data combined with video recordings.

#### 2.2.3 Selection of target muscles

Surface electromyographic signals were recorded from 10 target muscles on the primary force-generating side or dominant side, including seven lower-limb muscles and three trunk muscles. The lower-limb muscles included the tibialis anterior (TA), lateral gastrocnemius (LG), soleus (SOL), vastus medialis (VM), vastus lateralis (VL), biceps femoris (BF), and gluteus maximus (GM). The trunk muscles included the latissimus dorsi (LD), erector spinae (ES), and rectus abdominis (RA).

These muscles cover the key functional components of BBFT, including ankle stabilization, knee extension, hip extension, lower-limb braking support, trunk postural control, and pre-stroke body stability. Because the muscle-related data in this study were mainly used for trunk–lower limb time–frequency intermuscular coherence analysis, the analysis focused on the common oscillatory characteristics between trunk and lower-limb muscles to reflect neuromuscular coordination among trunk stability, lower-limb support, and stroke force-generation coupling during BBFT (Tong et al., 2023).

### 2.3 Testing procedures

After arriving at the experimental venue, participants first completed basic information registration, and their physical condition and eligibility for testing were confirmed by the researchers. All participants then performed a standardized warm-up following the same procedure, including low-intensity jogging, dynamic stretching, lower-limb activation exercises, trunk rotation exercises, shoulder, elbow, and wrist mobility exercises, and low-intensity badminton-specific footwork practice. The total warm-up duration was controlled within 10–15 min to ensure that participants reached a relatively stable state of motor readiness before formal testing.

After the warm-up, the skin over the target muscles was prepared using a standardized procedure, including shaving, light abrasion of the stratum corneum, and cleaning with 75% alcohol to reduce skin impedance and improve the quality of surface electromyographic signals. Surface electromyography electrodes were placed over the muscle belly along the direction of the muscle fibers and secured with elastic tape to minimize electrode displacement and movement artifacts during rapid movement and stroke execution in the badminton backhand footwork technique (BBFT). All electrodes were placed by the same researcher to reduce the influence of operator-related variability on signal acquisition.

Subsequently, infrared reflective markers were attached to the corresponding anatomical landmarks to establish an individualized three-dimensional lower-limb kinematic model. The markers were mainly placed on the pelvis, thigh, knee joint, shank, ankle joint, heel, and foot to obtain three-dimensional hip, knee, and ankle joint angles and angular velocities during BBFT. After marker placement, participants performed a static standing calibration trial to establish individualized body segment models and calibrate joint center positions.

Before formal testing, participants completed 5–10 familiarization BBFT trials to become accustomed to the testing environment, start signal, movement rhythm, rear-court backhand hitting area, and target landing area. Familiarization trials were not included in subsequent analyses. A programmable shuttle-feeding machine (SIBOASI S4025, Dongguan, China) was used for all familiarization and formal trials. Researcher-delivered feeding was not used during formal data collection. The machine was positioned on the longitudinal centerline of the opposite court, 1.0 m behind the short service line, with the shuttle-release outlet located 1.75 m above the court surface. The machine was fixed at a shuttle-release velocity of 12 m/s, a feeding elevation angle of 35°, and a feeding interval of 5 s. These parameters produced a standardized high and deep shuttle trajectory toward the participant’s rear-court backhand side.

The intended shuttle-feeding zone was a 1.0 m × 1.0 m rectangular area marked within the designated rear-court backhand region on the participant’s side of the court. The center of the feeding zone was located 1.0 m inside the back boundary line and 0.75 m inside the nearest singles sideline. Before each participant’s test, three calibration feeds were delivered to confirm that the shuttle followed the prescribed trajectory and entered the designated feeding zone. Minor machine adjustments were permitted only during this calibration procedure. Once calibration was completed, the machine position, release velocity, elevation angle, and feeding interval remained unchanged throughout the participant’s formal trials and were kept identical for the elite and amateur groups.

A feed was accepted when the shuttle crossed the net with the prescribed high trajectory and entered the predefined rear-court feeding zone. Feeds that contacted the net, landed outside the feeding zone, showed an evident deviation from the prescribed trajectory, or were released before the participant had established the standardized central ready position were rejected and repeated. Only trials involving an accepted standardized feed, completion of the prescribed BBFT sequence, and execution of a rear-court backhand clear were included in the analysis. Each participant completed 10 valid trials, with a 30–60 s recovery interval between consecutive trials.

During formal testing, participants started from the standardized central ready position. Each trial began when the shuttle was released by the shuttle-feeding machine. Participants moved toward the designated rear-court backhand area on the non-racket side and executed a rear-court backhand clear toward the predefined target area in the opponent’s rear court. The same machine position, release velocity, release height, trajectory, feeding interval, and rear-court feeding zone were used for all participants in the elite and amateur groups.

Trials were considered invalid, excluded from subsequent analyses, and repeated if any of the following occurred: incorrect movement initiation direction, obvious movement interruption, failure to reach the prescribed hitting area, incomplete backhand stroke execution, loss of balance, detachment of motion-capture markers, obvious abnormality in surface electromyographic signals, or synchronization failure. Testing continued until each participant completed 10 valid BBFT trials.

### 2.4 Movement cycle and phase segmentation

To ensure consistent temporal boundaries and clear biomechanical meaning for kinematic analysis and time–frequency intermuscular coherence analysis, the BBFT movement cycle was segmented based on infrared three-dimensional motion-capture data combined with video recordings. The BBFT analysis cycle was defined as the period from movement initiation at the central ready position to racket–shuttlecock contact. Movement initiation was defined as the instant when the support foot or moving foot began to clearly leave the ground, or when the body center of mass began to move toward the backhand side. Racket–shuttlecock contact was defined as the instant when the racket contacted the shuttlecock (McErlain-Naylor et al., 2020).

According to the technical structure and sport-specific functional characteristics of BBFT, the complete movement cycle was further divided into three phases: the movement initiation phase, braking support phase, and stroke force-generation phase. The movement initiation phase was defined as the period from movement initiation to before initial contact of the main support foot, mainly reflecting the ability to move rapidly from the central court position toward the rear-court backhand area. The braking support phase was defined as the period from initial contact of the main support foot to the completion of body center-of-mass braking and entry into the stroke preparation state, mainly reflecting lower-limb braking support, dynamic stability, and postural control. The stroke force-generation phase was defined as the period from the end of braking support to racket–shuttlecock contact, mainly reflecting coordination among lower-limb support, trunk control, and backhand stroke execution.

All key movement events were identified offline by two researchers based on motion-capture data and video recordings. After the movement cycle and phase boundaries of each valid trial were determined, three-dimensional kinematic data and surface electromyographic signals within the corresponding time windows were extracted and time-normalized to 0%–100% of the movement cycle for subsequent analyses of lower-limb joint coordination and trunk–lower limb time–frequency intermuscular coherence.

### 2.5 Data acquisition and processing

#### 2.5.1 Movement performance outcomes

Backhand footwork completion time and shot-placement accuracy were used to evaluate movement performance during the badminton backhand footwork technique (BBFT). Backhand footwork completion time was defined as the time interval from movement initiation to racket–shuttlecock contact, reflecting the speed with which participants-initiated movement from the central court position, moved to the rear-court backhand area, and coordinated the subsequent backhand stroke.

Shot-placement accuracy was used to evaluate the outcome of the standardized rear-court backhand clear performed during BBFT. Before testing, a predefined target area was established in the rear court on the opposite side of the net. A target hit was recorded when the shuttlecock crossed the net and landed within the predefined rear-court target area. A target miss was recorded when the shuttlecock crossed the net and landed within the valid court area but outside the target area. A trial was considered invalid and repeated if the shuttlecock hit the net or landed outside the valid court area, if the participant used a stroke other than the prescribed backhand clear, or if the footwork or stroke execution clearly deviated from the standardized task requirements (He et al., 2024).

Each participant completed 10 valid BBFT trials. The number of target hits among the 10 valid trials was recorded, and shot-placement accuracy was calculated as the percentage of target hits relative to the number of valid trials. The calculation formula was as follows:


$$Accuracy=\frac{N_{target}}{N_{valid}}\times 100\%$$

where $N_{target}$ represents the number of shots that landed within the target landing area, and $N_{valid}$ represents the number of valid BBFT trials. In the present study, $N_{valid}=10$.

#### 2.5.2 Three-dimensional kinematic data processing

Marker trajectory data collected using the infrared three-dimensional motion capture system were processed offline. First, the raw marker trajectories were inspected, and short gaps in marker trajectories were filled using interpolation. The three-dimensional coordinate data were then smoothed using a zero-phase low-pass filtering method to reduce the influence of high-frequency noise on the calculation of joint angles and angular velocities. The filter cut-off frequency was determined based on residual analysis and the quality of the kinematic data.

An individualized lower-limb segment model was established based on the static standing calibration data. Sagittal-plane hip, knee, and ankle joint angles and angular velocities of the main support side were calculated throughout the BBFT movement cycle. All kinematic data were time-normalized to the BBFT movement cycle and interpolated to 0%–100% of the movement cycle to ensure comparability across participants and trials.

This study focused on extracting hip, knee, and ankle joint angles and angular velocities of the main support side for subsequent lower-limb joint coordination analysis. The analyzed joint pairs included the hip–knee, hip–ankle, and knee–ankle joint pairs.

#### 2.5.3 Lower-limb joint coordination analysis

Continuous relative phase (CRP) was used to characterize the phase relationship between adjacent lower-limb joints throughout the BBFT movement cycle. Sagittal-plane hip, knee, and ankle joint angles and angular velocities of the main support side were first normalized. The phase angle of each joint was then calculated from its normalized angular displacement and angular velocity using the two-argument arctangent function. CRP was subsequently calculated as the difference between the phase angles of the proximal and distal joints and was wrapped to the range of −180° to 180° (Cowley and Gates, 2017; Du et al., 2023).

CRP was calculated separately for the hip–knee, hip–ankle, and knee–ankle joint pairs in each valid trial. Based on the resulting CRP values, each normalized point of the movement cycle was classified into one of four coordination patterns: in-phase (|CRP| ≤ 45°), anti-phase (|CRP| ≥ 135°), proximal-dominant (45° < CRP < 135°), or distal-dominant (−135° < CRP < −45°).

For each joint pair and trial, the number of normalized movement-cycle points assigned to each coordination pattern was divided by the total number of points in the movement cycle and expressed as a percentage. Consequently, each participant contributed ten trial-level percentage values for every joint-pair–coordination-pattern combination.

The coordination-pattern percentages were selected as the primary CRP-derived outcomes because the study aimed to compare the overall distribution of lower-limb coordination strategies across the complete BBFT movement cycle. These measures quantify the relative proportion of the movement performed using synchronized, opposing, proximal-dominant, or distal-dominant coordination. They were not intended to identify the precise temporal location of transitions between coordination states or group differences at individual normalized time points.

#### 2.5.4 Surface electromyographic signal processing and time–frequency intermuscular coherence analysis

MVC normalization was not applied because the present study did not compare amplitude-dependent EMG outcomes, such as RMS, integrated EMG, or peak activation, between participants or groups. The primary EMG-derived outcome was magnitude-squared intermuscular coherence, which is a dimensionless measure obtained by normalizing the squared cross-spectrum by the product of the two autospectra. Consequently, constant linear scaling of either EMG signal, including scaling to an MVC reference value, does not alter the resulting coherence coefficient. Therefore, MVC normalization was not required for the primary coherence analysis.

Nevertheless, signal acquisition and preprocessing were standardized across all participants. Electrode sites were prepared using the same skin-preparation procedure, electrodes were positioned over the muscle belly in alignment with the muscle-fiber direction by the same trained researcher, and identical filtering, rectification, smoothing, segmentation, and coherence-analysis parameters were applied to all participants, trials, and muscle pairs. Accordingly, the present coherence results should be interpreted as differences in frequency-specific common oscillatory coupling rather than differences in absolute muscle-activation amplitude.

Surface electromyographic signals were processed offline using a standardized signal-processing procedure. The raw EMG signals were acquired at a sampling frequency of 1000 Hz and band-pass filtered at 20–400 Hz using a fourth-order zero-phase Butterworth filter to reduce low-frequency movement artifacts and high-frequency noise. When evident power-line interference was detected, a 50-Hz notch filter was applied. The filtered signals were then full-wave rectified and smoothed using a 10-sample moving-average window, corresponding to 10 ms at the 1000-Hz sampling frequency.

Using the key movement events identified from the synchronized three-dimensional motion-capture and video data, the EMG signals from each valid BBFT trial were extracted from movement initiation to racket–shuttlecock contact. Each of the ten valid trials was processed separately. Therefore, the trial-level coherence outcomes were retained as repeated observations rather than being concatenated or averaged before inferential statistical analysis.

This study focused on time–frequency intermuscular coherence between the trunk and lower-limb muscles. The trunk muscles included the latissimus dorsi (LD), erector spinae (ES), and rectus abdominis (RA), whereas the lower-limb muscles included the tibialis anterior (TA), lateral gastrocnemius (LG), soleus (SOL), vastus medialis (VM), vastus lateralis (VL), biceps femoris (BF), and gluteus maximus (GM). Accordingly, 21 trunk–lower limb muscle pairs were analyzed: TA–LD, LG–LD, SOL–LD, VM–LD, VL–LD, BF–LD, GM–LD, TA–ES, LG–ES, SOL–ES, VM–ES, VL–ES, BF–ES, GM–ES, TA–RA, LG–RA, SOL–RA, VM–RA, VL–RA, BF–RA, and GM–RA.

Time–frequency intermuscular coherence was estimated using the short-time Fourier transform. The rectified and smoothed EMG signals from each trial were first divided into non-overlapping 500-sample segments, corresponding to 500 ms at the 1000-Hz sampling frequency. Any incomplete segment remaining at the end of a trial was not included in the segment-level analysis. The STFT-based coherence analysis followed the general approach described by Li and Sheng (2026) (Zhuang et al., 2026). All valid trials yielded the same number of complete 500-ms segments, and identical within-segment analysis settings were used across participants and groups.

Within each 500-ms segment, the signals were analyzed using a 200-sample Hamming window, corresponding to 200 ms. Consecutive windows overlapped by 150 samples, representing an overlap of 75%. The resulting window advancement was 50 samples, corresponding to a temporal step of 50 ms. The FFT length was equal to the window length at 200 points, producing a frequency-bin spacing of 5 Hz. The 200-ms window and 75% overlap were selected to provide repeated local spectral estimates within each 500-ms segment while balancing temporal localization and spectral characterization during the relatively short BBFT movement cycle.

The surrogate-corrected coherence maps obtained from all complete 500-ms segments within the same valid trial were averaged to produce a trial-level coherence map. Therefore, each valid trial contributed one averaged surrogate-corrected coherence map for every analyzed muscle pair.

The area of significant coherence (AZ) was used to quantify coherence within the predefined frequency bands. For each trial, the surrogate-corrected coherence values within each frequency band were integrated across time using the trapezoidal method and subsequently summed across the frequency bins included in that band. Separate AZ values were calculated for the α, β, and lower-γ frequency ranges. Consequently, each participant contributed ten trial-level AZ values for every muscle-pair–frequency-band combination.

The nominal frequency ranges were defined as α, 8–15 Hz; β, 15–30 Hz; and lower γ, 30–50 Hz. Because the FFT settings produced a frequency-bin spacing of 5 Hz, coherence was summarized using the available discrete frequency bins falling within these predefined ranges. Specifically, the α-band calculation included the 10- and 15-Hz bins; the β-band calculation included the 20-, 25-, and 30-Hz bins; and the lower-γ-band calculation included the 35-, 40-, 45-, and 50-Hz bins.

The upper limit of the lower-γ range was set at 50 Hz to restrict the analysis to lower-γ coupling and to avoid extending the analysis into higher-frequency surface-EMG components that are more susceptible to noise during rapid dynamic movement. When a 50-Hz notch filter was required because of evident power-line interference, the frequency bin directly affected by the notch filter was excluded from AZ integration to prevent filter-induced attenuation from being interpreted as a physiological difference.

AZ was retained as the primary coherence outcome because the present study aimed to compare the overall amount of significant frequency-band-specific trunk–lower limb coupling across the complete BBFT movement cycle. AZ integrates the magnitude and temporal extent of surrogate-corrected coherence within each predefined frequency range and provides a trial-level scalar outcome suitable for mixed-effects analysis. However, AZ was not intended to identify the precise temporal or spectral location of individual coherence regions.

The same preprocessing parameters, segmentation procedure, STFT settings, surrogate-correction procedure, coherence threshold, and frequency-band definitions were applied consistently across all participants, trials, and muscle pairs.

### 2.6 Statistical analysis

Statistical analyses of the participant characteristics presented in **Table 1** were performed using SPSS version 26.0 (IBM Corp., Armonk, NY, USA). Trial-level mixed-effects analyses were conducted using Python version 3.12.13. Continuous data are presented as mean ± standard deviation. For **Tables 2** and **3**, the ten trials were averaged within each participant and then summarized by group for descriptive presentation. All inferential analyses were conducted using the original trial-level observations.

**Table 2:** Mixed-effects model analysis of lower-limb joint coordination patterns during BBFT between the Elite and amateur groups (Unit: %).

Joint pair	Coordination pattern	Elite group	Amateur group	Estimated difference (95% CI)	F(df1, df2)	Raw P	FDR q
Hip-knee	In-phase	8.670 ± 2.104	7.121 ± 1.418	1.549 (0.328 to 2.770)	F(1, 29.79) = 6.71	0.015	0.025
Hip-knee	Anti-phase	59.526 ± 5.632	64.626 ± 3.458	-5.100 (-8.291 to -1.909)	F(1, 28.21) = 10.71	0.003	0.025
Hip-knee	Proximal-dominant	13.790 ± 1.636	13.381 ± 2.568	0.409 (-1.059 to 1.876)	F(1, 28.86) = 0.32	0.573	0.573
Hip-knee	Distal-dominant	18.014 ± 6.982	14.872 ± 5.511	3.142 (-1.126 to 7.409)	F(1, 32.26) = 2.25	0.144	0.157
Hip-ankle	In-phase	35.989 ± 11.633	29.535 ± 12.757	6.454 (-1.820 to 14.729)	F(1, 33.71) = 2.51	0.122	0.151
Hip-ankle	Anti-phase	11.869 ± 3.602	9.424 ± 5.517	2.445 (-0.729 to 5.620)	F(1, 29.27) = 2.48	0.126	0.151
Hip-ankle	Proximal-dominant	9.637 ± 4.699	6.378 ± 1.788	3.259 (0.800 to 5.718)	F(1, 21.82) = 7.56	0.012	0.025
Hip-ankle	Distal-dominant	42.505 ± 11.166	54.663 ± 14.685	-12.158 (-21.022 to -3.295)	F(1, 31.73) = 7.81	0.009	0.025
Knee-ankle	In-phase	5.539 ± 3.063	3.964 ± 2.146	1.575 (-0.224 to 3.374)	F(1, 30.45) = 3.19	0.084	0.126
Knee-ankle	Anti-phase	42.930 ± 14.359	31.634 ± 11.454	11.296 (2.484 to 20.108)	F(1, 32.40) = 6.81	0.014	0.025
Knee-ankle	Proximal-dominant	8.228 ± 2.717	5.470 ± 3.586	2.758 (0.598 to 4.918)	F(1, 31.68) = 6.77	0.014	0.025
Knee-ankle	Distal-dominant	43.303 ± 18.109	58.932 ± 14.489	-15.629 (-26.754 to -4.504)	F(1, 32.44) = 8.18	0.007	0.025

Data are presented as mean ± standard deviation. The between-group difference was calculated as elite minus amateur. Positive values indicate higher values in the elite group, whereas negative values indicate lower values in the elite group. Raw P represents the unadjusted P value, and FDR q represents the P value adjusted using the Benjamini–Hochberg false discovery rate procedure across the 12 comparisons. FDR q<0.05 was considered statistically significant. CI, confidence interval; FDR, false discovery rate.

**Table 3:** Mixed-effects model analysis of trunk–lower limb time–frequency intermuscular coherence during BBFT between the elite and amateur groups (Unit: Az).

Muscle pair	Frequency band	Elite group	Amateur group	Estimated difference (95% CI)	F(df1, df2)	Raw P	FDR q
TA-LD	α	0.023000 ± 0.030000	0.017000 ± 0.030000	0.006000 (-0.014311 to 0.026311)	F(1, 33.99) = 0.36	0.552	0.610
TA-LD	β	0.047000 ± 0.052000	0.023000 ± 0.039000	0.024000 (-0.007465 to 0.055465)	F(1, 31.29) = 2.42	0.130	0.190
TA-LD	γ	0.044000 ± 0.053000	0.000228 ± 0.000049	0.043772 (0.017528 to 0.070017)	F(1, 17.00) = 12.38	0.003	0.098
LG-LD	α	0.020000 ± 0.029000	0.004000 ± 0.005000	0.016000 (0.001424 to 0.030576)	F(1, 18.01) = 5.32	0.033	0.118
LG-LD	β	0.029000 ± 0.047000	0.001000 ± 0.003000	0.028000 (0.004578 to 0.051422)	F(1, 17.14) = 6.35	0.022	0.099
LG-LD	γ	0.023000 ± 0.033000	0.000259 ± 0.000066	0.022741 (0.006334 to 0.039149)	F(1, 17.00) = 8.55	0.009	0.098
SOL-LD	α	0.020000 ± 0.041000	0.000350 ± 0.000048	0.019650 (-0.000691 to 0.039990)	F(1, 17.00) = 4.15	0.057	0.129
SOL-LD	β	0.025000 ± 0.039000	0.000308 ± 0.000059	0.024692 (0.005391 to 0.043993)	F(1, 16.99) = 7.29	0.015	0.098
SOL-LD	γ	0.007000 ± 0.013000	0.000212 ± 0.000054	0.006788 (0.000304 to 0.013272)	F(1, 17.00) = 4.88	0.041	0.118
VM-LD	α	0.015000 ± 0.020000	0.006000 ± 0.010000	0.009000 (-0.001865 to 0.019865)	F(1, 24.97) = 2.91	0.100	0.178
VM-LD	β	0.014000 ± 0.019000	0.002000 ± 0.005000	0.012000 (0.002375 to 0.021625)	F(1, 19.38) = 6.79	0.017	0.098
VM-LD	γ	0.019000 ± 0.031000	0.004000 ± 0.011000	0.015000 (-0.001047 to 0.031047)	F(1, 21.19) = 3.77	0.065	0.142
VL-LD	α	0.011000 ± 0.022000	0.002000 ± 0.006000	0.009000 (-0.002276 to 0.020276)	F(1, 19.49) = 2.78	0.111	0.178
VL-LD	β	0.009000 ± 0.015000	0.000222 ± 0.000067	0.008778 (0.001293 to 0.016263)	F(1, 17.00) = 6.12	0.024	0.102
VL-LD	γ	0.004000 ± 0.007000	0.000295 ± 0.000065	0.003705 (0.000211 to 0.007200)	F(1, 17.00) = 5.01	0.039	0.118
BF-LD	α	0.015000 ± 0.020000	0.002000 ± 0.002000	0.013000 (0.002954 to 0.023046)	F(1, 17.33) = 7.43	0.014	0.098
BF-LD	β	0.012000 ± 0.014000	0.001000 ± 0.001000	0.011000 (0.004028 to 0.017972)	F(1, 17.17) = 11.06	0.004	0.098
BF-LD	γ	0.006000 ± 0.008000	0.000354 ± 0.000092	0.005646 (0.001666 to 0.009627)	F(1, 17.00) = 8.96	0.008	0.098
GM-LD	α	0.022000 ± 0.031000	0.041000 ± 0.023000	0.019000 (-0.037545 to 0.000455)	F(1, 31.38) = 4.36	0.045	0.123
GM-LD	β	0.017000 ± 0.026000	0.044000 ± 0.061000	-0.027000 (-0.059337 to 0.005337)	F(1, 23.05) = 2.98	0.098	0.178
GM-LD	γ	0.014000 ± 0.029000	0.015000 ± 0.016000	-0.001000 (-0.016913 to 0.014913)	F(1, 26.33) = 0.02	0.898	0.928
TA-ES	α	0.026000 ± 0.048000	0.000338 ± 0.000080	0.025662 (0.001833 to 0.049491)	F(1, 17.00) = 5.16	0.036	0.118
TA-ES	β	0.031000 ± 0.062000	0.000237 ± 0.000061	0.030763 (-0.000147 to 0.061674)	F(1, 17.00) = 4.41	0.051	0.129
TA-ES	γ	0.015000 ± 0.040000	0.000206 ± 0.000037	0.014794 (-0.005032 to 0.034620)	F(1, 17.00) = 2.48	0.134	0.192
LG-ES	α	0.033000 ± 0.046000	0.005000 ± 0.012000	0.028000 (0.004507 to 0.051493)	F(1, 19.29) = 6.21	0.022	0.099
LG-ES	β	0.029000 ± 0.039000	0.000198 ± 0.000056	0.028802 (0.009412 to 0.048191)	F(1, 17.00) = 9.82	0.006	0.098
LG-ES	γ	0.014000 ± 0.036000	0.000314 ± 0.000096	0.013686 (-0.003906 to 0.031277)	F(1, 16.96) = 2.70	0.119	0.182
SOL-ES	α	0.008000 ± 0.011000	0.001000 ± 0.002000	0.007000 (0.001475 to 0.012525)	F(1, 18.12) = 7.08	0.016	0.098
SOL-ES	β	0.003000 ± 0.007000	0.000198 ± 0.000040	0.002802 (-0.000682 to 0.006286)	F(1, 17.00) = 2.88	0.108	0.178
SOL-ES	γ	0.001000 ± 0.001000	0.001000 ± 0.001000	<0.000001 (-0.000677 to 0.000677)	F(1, 33.99) = 0.00	1.000	1.000
VM-ES	α	0.013000 ± 0.018000	0.002000 ± 0.004000	0.011000 (0.001836 to 0.020164)	F(1, 18.65) = 6.33	0.021	0.099
VM-ES	β	0.011000 ± 0.024000	0.000307 ± 0.000037	0.010693 (-0.001299 to 0.022686)	F(1, 17.00) = 3.54	0.077	0.157
VM-ES	γ	0.005000 ± 0.012000	0.000205 ± 0.000049	0.004795 (-0.001140 to 0.010731)	F(1, 17.00) = 2.91	0.107	0.178
VL-ES	α	0.001000 ± 0.001000	0.001000 ± 0.001000	<0.000001 (-0.000678 to 0.000678)	F(1, 34.00) = 0.00	1.000	1.000
VL-ES	β	0.004000 ± 0.013000	0.000200 ± 0.000051	0.003800 (-0.002691 to 0.010290)	F(1, 17.00) = 1.53	0.234	0.283
VL-ES	γ	0.001000 ± 0.002000	0.000356 ± 0.000089	0.000644 (-0.000350 to 0.001638)	F(1, 17.06) = 1.87	0.190	0.234
BF-ES	α	0.016000 ± 0.038000	0.000271 ± 0.000085	0.015729 (-0.003269 to 0.034727)	F(1, 17.00) = 3.05	0.099	0.178
BF-ES	β	0.018000 ± 0.040000	0.000310 ± 0.000043	0.017690 (-0.002164 to 0.037545)	F(1, 17.00) = 3.53	0.077	0.157
BF-ES	γ	0.006000 ± 0.012000	0.000221 ± 0.000064	0.005779 (-0.000196 to 0.011754)	F(1, 17.00) = 4.16	0.057	0.129
GM-ES	α	0.007000 ± 0.011000	0.010000 ± 0.014000	-0.003000 (-0.011536 to 0.005536)	F(1, 31.98) = 0.51	0.479	0.539
GM-ES	β	0.025000 ± 0.045000	0.005000 ± 0.009000	0.020000 (-0.002676 to 0.042676)	F(1, 18.36) = 3.42	0.080	0.158
GM-ES	γ	0.010000 ± 0.016000	0.011000 ± 0.026000	-0.001000 (-0.015741 to 0.013741)	F(1, 28.19) = 0.02	0.891	0.928
TA-RA	α	0.017000 ± 0.033000	0.004000 ± 0.007000	0.013000 (-0.003787 to 0.029787)	F(1, 18.49) = 2.64	0.121	0.182
TA-RA	β	0.012000 ± 0.028000	0.003000 ± 0.005000	0.009000 (-0.004849 to 0.022849)	F(1, 18.08) = 1.86	0.189	0.234
TA-RA	γ	0.018000 ± 0.052000	0.000290 ± 0.000077	0.017710 (-0.008100 to 0.043519)	F(1, 17.00) = 2.10	0.166	0.221
LG-RA	α	0.027000 ± 0.041000	0.001000 ± 0.002000	0.026000 (0.005383 to 0.046617)	F(1, 17.08) = 7.07	0.016	0.098
LG-RA	β	0.011000 ± 0.019000	0.000215 ± 0.000066	0.010785 (0.001376 to 0.020194)	F(1, 17.00) = 5.85	0.027	0.107
LG-RA	γ	0.005000 ± 0.009000	0.000292 ± 0.000039	0.004708 (0.000233 to 0.009182)	F(1, 17.00) = 4.93	0.040	0.118
SOL-RA	α	0.005000 ± 0.008000	0.001000 ± 0.002000	0.004000 (-0.000077 to 0.008077)	F(1, 19.11) = 4.21	0.054	0.129
SOL-RA	β	0.009000 ± 0.024000	0.000198 ± 0.000056	0.008802 (-0.003170 to 0.020774)	F(1, 17.00) = 2.41	0.139	0.195
SOL-RA	γ	0.001000 ± 0.004000	0.000291 ± 0.000050	0.000709 (-0.001280 to 0.002698)	F(1, 17.01) = 0.57	0.462	0.529
VM-RA	α	0.010000 ± 0.015000	0.000312 ± 0.000080	0.009688 (0.002223 to 0.017154)	F(1, 17.00) = 7.50	0.014	0.098
VM-RA	β	0.006000 ± 0.017000	0.000318 ± 0.000067	0.005682 (-0.002649 to 0.014013)	F(1, 16.99) = 2.07	0.168	0.221
VM-RA	γ	0.011000 ± 0.033000	0.000285 ± 0.000072	0.010715 (-0.005783 to 0.027214)	F(1, 17.00) = 1.88	0.188	0.234
VL-RA	α	0.008000 ± 0.019000	0.000272 ± 0.000061	0.007728 (-0.001731 to 0.017187)	F(1, 17.00) = 2.97	0.103	0.178
VL-RA	β	0.002000 ± 0.005000	0.000206 ± 0.000060	0.001794 (-0.000688 to 0.004275)	F(1, 17.00) = 2.33	0.146	0.200
VL-RA	γ	0.001000 ± 0.004000	0.000233 ± 0.000068	0.000767 (-0.001223 to 0.002756)	F(1, 17.01) = 0.66	0.427	0.499
BF-RA	α	0.007000 ± 0.015000	0.001000 ± 0.003000	0.006000 (-0.001560 to 0.013560)	F(1, 18.38) = 2.77	0.113	0.178
BF-RA	β	0.021000 ± 0.041000	0.001000 ± 0.001000	0.020000 (-0.000387 to 0.040387)	F(1, 17.02) = 4.28	0.054	0.129
BF-RA	γ	0.005000 ± 0.009000	0.000291 ± 0.000054	0.004709 (0.000239 to 0.009179)	F(1, 17.00) = 4.94	0.040	0.118
GM-RA	α	0.016000 ± 0.032000	0.009000 ± 0.011000	0.007000 (-0.009702 to 0.023702)	F(1, 20.83) = 0.76	0.393	0.467
GM-RA	β	0.014000 ± 0.022000	0.012000 ± 0.017000	0.002000 (-0.011348 to 0.015348)	F(1, 31.93) = 0.09	0.762	0.814
GM-RA	γ	0.006000 ± 0.014000	0.008000 ± 0.008000	-0.002000 (-0.009795 to 0.005795)	F(1, 27.05) = 0.28	0.603	0.655

Data are presented as mean ± standard deviation. Values are reported to six decimal places to distinguish very small positive AZ values from exact zeros. The between-group difference was calculated as elite minus amateur. Positive values indicate higher AZ values in the elite group, whereas negative values indicate lower AZ values in the elite group. Raw P represents the unadjusted P value, and FDR q represents the P value adjusted using the Benjamini–Hochberg false discovery rate procedure across the 63 muscle-pair–frequency-band comparisons. FDR q<0.05 was considered statistically significant. AZ, area of significant coherence; CI, confidence interval; FDR, false discovery rate. Denominator degrees of freedom were obtained using a Satterthwaite-type approximation; values close to 17 reflect the effective information for the group contrast under strongly unequal group variances, with uncertainty dominated by one group of 18 participants.

Trial-level coordination outcomes included four coordination patterns for the hip–knee, hip–ankle, and knee–ankle joint pairs, resulting in 12 outcomes. Intermuscular coherence outcomes included AZ values for 21 muscle pairs in the α, β, and γ frequency bands, resulting in 63 outcomes.

Between-group differences were examined using linear mixed-effects models. Group, trial, and the group-by-trial interaction were included as fixed effects, with participant included as a random intercept. Trial was treated as a categorical repeated factor, and a first-order autoregressive covariance structure was used to model correlations between consecutive trials. Group-specific variance components were permitted. Models were estimated using restricted maximum likelihood, with denominator degrees of freedom calculated using a Satterthwaite-type approximation.

The primary contrast was the estimated marginal mean difference between the elite and amateur groups across the ten trials. Differences were calculated as elite minus amateur and are reported with 95% confidence intervals, F statistics, denominator degrees of freedom, and two-tailed P values.

Model assumptions were evaluated using residual histograms, normal quantile–quantile plots, Shapiro–Wilk tests, and residual-versus-fitted plots. Diagnostic plots are provided in the **Supplementary Material**. Robustness was additionally assessed using 10,000 participant-cluster bootstrap resamples, with all ten trials retained together for each resampled participant.

The AZ data were also examined for exact and near-zero values. None of the 22,680 observations was exactly zero, although 7,553 values were below 0.0005 and would appear as 0.000 when rounded to three decimal places. Therefore, these entries represent small positive values rather than genuine zeros.

Benjamini–Hochberg false discovery rate correction was applied separately to the 12 coordination comparisons and the 63 coherence comparisons. Both raw P values and FDR-adjusted P values (q values) are reported. Statistical significance was defined as q<0.05 (Lytsy, 2018). Although adjustment for years of badminton training was proposed in our earlier revision, this covariate was not included in the final mixed-effects models. The primary objective was to compare the overall coordination characteristics of elite and amateur players within their existing training backgrounds, rather than to estimate between-group differences at an equivalent duration of training. Training experience was considered an important component of the background associated with competitive level; adjusting for it would therefore address a different research question. Years of training were reported descriptively in **Table 1**, and the group contrasts should be interpreted as differences between competitive-level groups without adjustment for training duration, rather than as associations independent of training experience.

## 3 Results

### 3.1 Comparison of lower-limb joint coordination patterns

All 12 coordination models and all 63 coherence models converged successfully. Shapiro–Wilk tests indicated residual normality deviations in four of the 12 coordination models and all 63 AZ models. Complete residual histograms, normal Q–Q plots, residuals-versus-fitted plots, and trial-level group distributions are provided in the **Supplementary Material**. Participant-cluster bootstrap sensitivity analyses supported the seven FDR-significant coordination findings but did not alter the absence of FDR-significant coherence findings. The four coordination outcomes showing departures from residual normality were hip–ankle anti-phase coordination, hip–ankle proximal-dominant coordination, knee–ankle in-phase coordination, and knee–ankle proximal-dominant coordination (all Shapiro–Wilk P<0.05).

As shown in **Table 2**, the mixed-effects models revealed significant between-group differences in several lower-limb coordination patterns during BBFT after Benjamini–Hochberg false discovery rate correction.

For the hip–knee joint pair, the elite group showed a significantly higher complete-cycle percentage of in-phase coordination than the amateur group, with an estimated between-group difference of 1.549 percentage points (95% CI: 0.328 to 2.770; F(1, 29.79) = 6.71, raw P = 0.015, FDR q = 0.025). In contrast, the elite group showed a significantly lower percentage of anti-phase coordination, with an estimated difference of −5.100 percentage points (95% CI: −8.291 to −1.909; F(1, 28.21) = 10.71, raw P = 0.003, FDR q = 0.025). No significant between-group differences were observed for proximal-dominant coordination (estimated difference = 0.409 percentage points, 95% CI: −1.059 to 1.876; F(1, 28.86) = 0.32, raw P = 0.573, FDR q = 0.573) or distal-dominant coordination (estimated difference = 3.142 percentage points, 95% CI: −1.126 to 7.409; F(1, 32.26) = 2.25, raw P = 0.144, FDR q = 0.157).

For the hip–ankle joint pair, the elite group showed a significantly higher percentage of proximal-dominant coordination than the amateur group, with an estimated difference of 3.259 percentage points (95% CI: 0.800 to 5.718; F(1, 21.82) = 7.56, raw P = 0.012, FDR q = 0.025). The elite group also showed a significantly lower percentage of distal-dominant coordination, with an estimated difference of −12.158 percentage points (95% CI: −21.022 to −3.295; F(1, 31.73) = 7.81, raw P = 0.009, FDR q = 0.025). No significant between-group differences were observed for in-phase coordination (estimated difference = 6.454 percentage points, 95% CI: −1.820 to 14.729; F(1, 33.71) = 2.51, raw P = 0.122, FDR q = 0.151) or anti-phase coordination (estimated difference = 2.445 percentage points, 95% CI: −0.729 to 5.620; F(1, 29.27) = 2.48, raw P = 0.126, FDR q = 0.151).

For the knee–ankle joint pair, the elite group showed significantly higher percentages of anti-phase coordination (estimated difference = 11.296 percentage points, 95% CI: 2.484 to 20.108; F(1, 32.40) = 6.81, raw P = 0.014, FDR q = 0.025) and proximal-dominant coordination (estimated difference = 2.758 percentage points, 95% CI: 0.598 to 4.918; F(1, 31.68) = 6.77, raw P = 0.014, FDR q = 0.025) than the amateur group. Conversely, the elite group showed a significantly lower percentage of distal-dominant coordination, with an estimated difference of −15.629 percentage points (95% CI: −26.754 to −4.504; F(1, 32.44) = 8.18, raw P = 0.007, FDR q = 0.025). No significant between-group difference was observed for knee–ankle in-phase coordination (estimated difference = 1.575 percentage points, 95% CI: −0.224 to 3.374; F(1, 30.45) = 3.19, raw P = 0.084, FDR q = 0.126).

Overall, seven coordination-pattern comparisons remained statistically significant after FDR correction: hip–knee in-phase and anti-phase coordination; hip–ankle proximal-dominant and distal-dominant coordination; and knee–ankle anti-phase, proximal-dominant, and distal-dominant coordination.

### 3.2 Comparison of trunk–lower limb time–frequency intermuscular coherence

As shown in **Figure 1** and **Table 3**, several muscle-pair–frequency-band comparisons showed nominal between-group differences before correction for multiple comparisons. Specifically, 23 of the 63 comparisons had raw P values below 0.05. However, none remained statistically significant after Benjamini–Hochberg false discovery rate correction across the 63 comparisons (all FDR q≥0.098).

**Figure 1:**
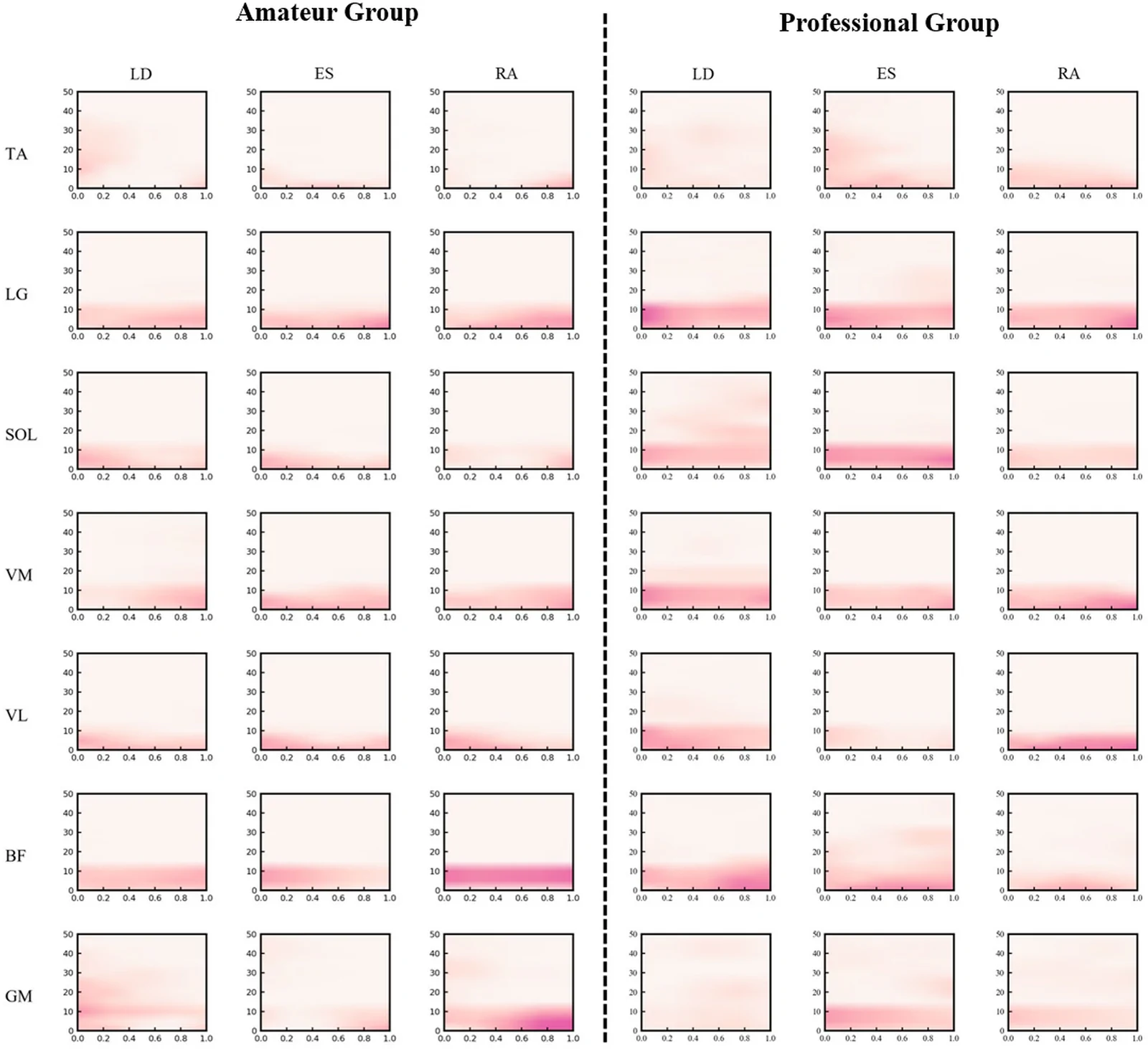
Time–frequency intermuscular coherence maps of trunk–lower limb muscle pairs during BBFT in the elite and amateur groups.

The smallest raw P value was observed for TA–LD coherence in the γ band. The elite group showed a higher AZ value than the amateur group, with a between-group difference of 0.043772 (95% CI: 0.017528 to 0.070017; F(1, 17.00) = 12.38, raw P = 0.003); however, this difference did not remain significant after FDR correction (FDR q = 0.098). Therefore, no LD-, ES-, or RA-related muscle-pair–frequency-band comparison was considered statistically significant after correction for multiple comparisons.

## 4 Discussion

The present study compared CRP-derived lower-limb coordination-pattern distributions and frequency-band-specific trunk–lower limb intermuscular coherence between elite and amateur badminton players during a standardized BBFT–backhand-clear task. After Benjamini–Hochberg FDR correction, seven lower-limb coordination-pattern comparisons remained statistically significant. Specifically, the elite group demonstrated a higher hip–knee in-phase proportion, a lower hip–knee anti-phase proportion, higher proximal-dominant coordination in the hip–ankle and knee–ankle joint pairs, lower distal-dominant coordination in these joint pairs, and a higher knee–ankle anti-phase proportion. In contrast, none of the 63 muscle-pair–frequency-band coherence comparisons remained statistically significant after FDR correction. Therefore, the principal findings concern differences in complete-cycle lower-limb coordination-pattern distributions rather than confirmed differences in trunk–lower limb intermuscular coherence.

For the hip–knee joint pair, the elite group showed a higher proportion of in-phase coordination and a lower proportion of anti-phase coordination across the complete BBFT cycle. This pattern indicates that the hip and knee spent a greater proportion of the movement cycle in similar phase relationships and a smaller proportion in opposing phase relationships in the elite group. During rear-court backhand footwork, players must combine rapid backward-lateral displacement with braking, postural support, and preparation for the backhand clear. A greater overall proportion of hip–knee in-phase coordination may reflect a more consistent organization of these linked lower-limb actions. This interpretation is compatible with evidence that badminton-specific balance and footwork training can improve dynamic stability and movement performance, although the present cross-sectional findings cannot establish that training caused the observed coordination differences (Malwanage et al., 2022).

For the hip–ankle joint pair, the elite group showed a higher proximal-dominant proportion and a lower distal-dominant proportion. This result suggests that the hip more frequently led the phase relationship between these joints, whereas the amateur group relied more heavily on ankle-led adjustments. The hip contributes to control of the center of mass, backward-lateral displacement, pelvic orientation, and preparation for trunk rotation. A greater proximal contribution may reduce the need for repeated distal corrections at the ankle when entering the rear-court support position. Previous badminton research has associated core-strength development with dynamic balance and agility, supporting the broader importance of proximal control during rapid court movement (Ozmen and Aydogmus, 2016). Nevertheless, the present CRP outcomes represent coordination-state percentages and do not directly measure hip strength, joint moments, or force production.

The knee–ankle results showed a related but more differentiated pattern. The elite group demonstrated higher anti-phase and proximal-dominant proportions and a lower distal-dominant proportion. In a rapid braking task, a higher anti-phase proportion should not automatically be interpreted as poorer coordination. Instead, it may represent functional differentiation between the knee and ankle, with the knee contributing to braking and regulation of body height while the ankle controls foot placement and ground-contact stability. The simultaneous increase in proximal-dominant coordination and decrease in distal-dominant coordination further suggest that the elite group relied less on ankle-led correction. Experience-related differences in inter-joint organization have also been reported during rapid cutting movements, in which coordination strategies vary according to motor experience and task demands (Pan et al., 2024). The present findings extend this concept to standardized badminton rear-court backhand footwork.

Taken together, the CRP findings suggest that the elite group used a complete-cycle coordination strategy characterized by greater hip–knee synchronization and greater proximal contribution to hip–ankle and knee–ankle organization. These outcomes provide information that cannot be obtained from movement time, isolated peak joint angles, or single-joint range-of-motion measures alone. Wearable-sensor research in badminton has demonstrated the value of movement-specific measurements for characterizing training load and court actions, but these measures do not directly describe how adjacent joints are organized relative to one another (Liu et al., 2024). The present CRP-derived outcomes therefore provide complementary evidence regarding overall inter-joint coordination strategies. However, because the analysis was based on complete-cycle percentages, it cannot determine the precise movement phase in which the observed differences occurred.

The intermuscular coherence findings should be interpreted cautiously because none of the 63 comparisons remained statistically significant after FDR correction. Although 23 of the 63 muscle-pair–frequency-band comparisons had raw P values below 0.05, none remained statistically significant after FDR correction. The smallest adjusted value was q=0.098. Thus, the present results do not provide corrected statistical evidence that elite and amateur players differed in any specific LD-, ES-, or RA-related muscle-pair–frequency-band AZ outcome. The nominal differences may be useful for generating hypotheses, but they should not be interpreted as confirmed evidence of stronger or more efficient trunk–lower limb neuromuscular coupling in elite players.

This absence of FDR-corrected coherence findings does not necessarily imply that trunk–lower limb coupling is physiologically unimportant during BBFT. Rather, it indicates that the current dataset could not distinguish the observed pair- and frequency-specific differences from the number of effects expected when testing 63 comparisons. Badminton lunge studies have demonstrated that movement direction produces distinct mechanical demands on the lower-limb joints (Yu et al., 2021), while sensor-based research in racket sports has emphasized that stroke execution is embedded within the movement actions that precede and accompany it (Perri et al., 2022). These studies support the relevance of examining task-specific neuromuscular organization, but they do not provide a basis for treating the present uncorrected coherence differences as established findings.

Similarly, coordinated activity between trunk and lower-limb muscles has been observed during dynamic landing-to-jump movements requiring rapid transitions among impact absorption, postural control, and subsequent force generation (Iida et al., 2012). BBFT may involve comparable requirements for integrating distal support with proximal stabilization. However, because none of the present coherence comparisons survived FDR correction, specific functional interpretations involving TA–LD, BF–LD, SOL–ES, or other individual muscle pairs would be premature. Such pair-specific interpretations should be examined in future confirmatory studies with larger samples and prespecified muscle-pair hypotheses.

The frequency-band findings also warrant caution. Nominal group differences occurred across α, β, and γ bands, but no frequency-band-specific comparison remained significant after correction. Consequently, the results do not support the conclusion that elite players demonstrate enhanced coupling within any particular frequency band. Furthermore, the physiological meaning of surface-EMG intermuscular coherence is task dependent, and numerical frequency boundaries should not be assigned a single neural origin without additional evidence (Zhang et al., 2026). The current AZ results should therefore be viewed as exploratory observations of common oscillatory coupling rather than direct evidence of specific cortical or subcortical pathways.

The task-specific nature of BBFT further limits interpretation. Biomechanical comparisons of forehand and backhand lunges have shown that movement direction alters lower-limb joint demands and loading strategies (Xie et al., 2025). The present task involved backward-lateral movement toward the non-racket-side rear court, establishment of a support position, and preparation for a backhand clear. Therefore, the observed CRP findings should not be generalized automatically to forward lunges, forehand footwork, jump smashes, or other badminton techniques. The absence of corrected coherence findings may also partly reflect the variability and complexity of the combined footwork-and-stroke task.

Evidence from other explosive sport actions suggests that skilled performance depends on task-specific organization rather than uniform increases in the activity or coupling of all muscles. For example, muscle-synergy research in basketball shooting has shown that successful performance can depend on structured contributions from selected muscles (Matsunaga and Oshikawa, 2022). Although muscle synergy and intermuscular coherence are distinct analytical constructs, both approaches highlight the complexity of neuromuscular organization. In the present study, however, the FDR-corrected findings support structured differences in joint coordination but do not confirm corresponding differences in individual intermuscular coherence outcomes.

The coordination characteristics observed in the elite group may have practical relevance for BBFT training. Greater proximal-dominant coordination in the hip–ankle and knee–ankle joint pairs suggests that training exercises emphasizing proximal control, braking, and coordinated hip–knee–ankle positioning may be worthy of further investigation. However, CRP pattern percentages do not directly measure movement efficiency, joint loading, tissue stress, or injury risk. Therefore, the observed coordination patterns should be considered potential targets for future intervention studies rather than established indicators of superior performance or injury protection.

The AZ audit also has important implications for interpretation. None of the 22,680 trial-level AZ observations was exactly zero, but 7,553 values were below 0.0005 and would appear as 0.000 when rounded to three decimal places. Thus, the previously observed zero-like values represented very small positive values rather than genuine absence of coherence. Nevertheless, the strong concentration of near-zero values, particularly in some amateur-group outcomes, indicates a pronounced lower-bound distribution. In addition, residual diagnostics demonstrated deviations from normality across the AZ models. Although heterogeneous variance components and participant-cluster bootstrap analyses were used to evaluate robustness, these distributional characteristics reinforce the need for caution when interpreting the uncorrected coherence comparisons.

Overall, the revised findings demonstrate competitive-level differences in selected complete-cycle lower-limb coordination-pattern distributions but do not demonstrate FDR-corrected differences in trunk–lower limb intermuscular coherence. The results therefore support a more focused interpretation than the original analysis: elite and amateur players differed in how adjacent lower-limb joints were organized across the complete BBFT cycle, whereas evidence for muscle-pair- and frequency-specific coherence differences remained inconclusive. Given the cross-sectional design, the observed coordination differences may reflect long-term training adaptation, pre-existing neuromuscular characteristics, talent-selection effects, or a combination of these factors.

### 4.1 Study limitations

Several limitations should be considered. First, only male badminton players were included, limiting generalizability to female players. Second, the standardized laboratory-based BBFT task did not reproduce the perceptual, tactical, and environmental variability of competition. Third, participants were tested under non-fatigued conditions; therefore, the findings may not apply to fatigued training or match situations.

Fourth, the cross-sectional design precludes causal inference. Differences between the elite and amateur groups may reflect training adaptation, talent selection, pre-existing neuromuscular characteristics, or their combination. Years of training alone also cannot fully represent training volume, intensity, coaching quality, or competitive experience.

Fifth, CRP waveforms and time–frequency coherence maps were summarized as coordination-pattern percentages and frequency-band-specific AZ values. These scalar measures characterize complete-cycle outcomes but do not preserve the temporal sequence of coordination or localize differences within the time–frequency domain. Short-duration or phase-specific effects may therefore have been overlooked.

Sixth, a substantial proportion of AZ observations was concentrated near zero, and residual diagnostics indicated non-normality in the coherence models. Although heterogeneous variance components and participant-cluster bootstrap sensitivity analyses were applied, these distributional characteristics may reduce the precision and reliability of conventional model-based inference.

Finally, the sample comprised only 36 participants, providing limited statistical power after correction for 63 coherence comparisons. Larger longitudinal studies incorporating female players, fatigue conditions, competition-like tasks, joint kinetics, and waveform- or cluster-based analyses are required to confirm the coordination findings and determine whether reproducible intermuscular coherence differences exist.

Furthermore, years of badminton training differed substantially between groups and were not included as a covariate in the final models. Consequently, the observed group differences cannot be separated from differences in training duration and should not be interpreted as associations with competitive level that are independent of training experience.

## 5 Conclusion

This cross-sectional study identified significant differences between elite and amateur male badminton players in selected complete-cycle lower-limb coordination-pattern distributions during a standardized rear-court BBFT–backhand-clear task. Compared with the amateur group, the elite group demonstrated a higher hip–knee in-phase proportion, a lower hip–knee anti-phase proportion, higher proximal-dominant and lower distal-dominant coordination in the hip–ankle and knee–ankle joint pairs, and a higher knee–ankle anti-phase proportion.

These findings indicate different overall lower-limb coordination strategies between the two competitive-level groups, characterized by greater proximal contribution in the elite group. However, they do not identify the specific movement phases in which these differences occurred and should not be interpreted as direct evidence of improved performance, reduced joint loading, or injury prevention.

Although several intermuscular coherence comparisons showed raw P values below 0.05, none remained statistically significant after FDR correction across the 63 comparisons. Therefore, the present study does not provide corrected statistical evidence of muscle-pair- or frequency-specific differences in trunk–lower limb intermuscular coherence between the elite and amateur groups.

The findings provide preliminary, task-specific reference information for future research on badminton lower-limb coordination. Longitudinal and intervention studies with larger and more diverse samples, fatigue and competition-like conditions, and waveform- or cluster-based analyses are required before the identified coordination characteristics can be considered targets for movement optimization, training prescription, or injury-prevention strategies.

## Data Availability

The original contributions presented in the study are included in the article/**Supplementary Material**. Further inquiries can be directed to the corresponding author.
